# Quantitative elucidation of associations between nucleotide identity and physicochemical properties of amino acids and the functional insight

**DOI:** 10.1016/j.csbj.2021.07.012

**Published:** 2021-07-17

**Authors:** Yan-Ting Jin, Tian-Yue Jin, Zhi-Li Zhang, Yuan-Nong Ye, Zixin Deng, Ju Wang, Feng-Biao Guo

**Affiliations:** a School of Life Science and Technology, University of Electronic Science and Technology of China, 611731 Chengdu, China; b Key Laboratory of Combinatorial Biosynthesis and Drug Discovery, Ministry of Education and School of Pharmaceutical Sciences, Wuhan University, 430071 Wuhan, China; c Department of Medical Informatics, Bioinformatics and BioMedical Bigdata Mining Laboratory, School of Big Health, Guizhou Medical University, 550025 Guiyang, China; d School of Biomedical Engineering, Tianjin Medical University, 300070 Tianjin, China

**Keywords:** Codon-amino acid association, Nucleotide combination at specific codon position, Amino acid physicochemical property, A_2_ versus T_2_ frequency, The hydropathy and GRAVY value, Informational versus operational functions

## Abstract

Studies on codon property would deepen our understanding of the origin of primitive life and enlighten biotechnical application. Here, we proposed a quantitative measurement of codon-amino acid association and found that seven out of 13 physicochemical properties have stronger associations with the nucleotide identity at the second codon position, indicating that protein structure and function may associate more closely with it than the other two sites. When extending the effect of codon-amino acid association to protein level, it was found that the correlation between the second codon position (measured by the relative frequencies of nucleobase T and A at this codon site) and hydrophobicity (by the form of GRAVY value) became stronger with 96% genomes having R > 0.90 and p < 1e-60. Furthermore, we revealed that informational genes encoding proteins have lower GRAVY values than operational proteins (p < 3e-37) in both prokaryotic and eukaryotic genomes. The above results reveal a complete link from codon identity (A_2_ versus T_2_) to amino acid property (hydrophilic versus hydrophobic) and then to protein functions (informational versus operational). Hence, our work may help to understand how the nucleotide sequence determines protein function.

## Graphical abstract

**Figure:**
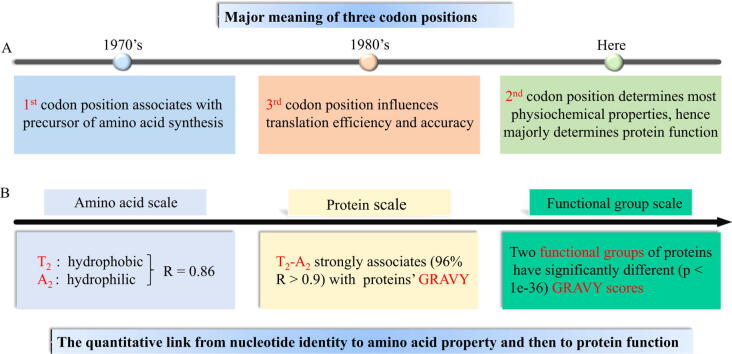

## 1 Introduction

Codon property has attracted the attention of many researchers [1]. Early in the 1980s, it was found that synonymous codons were not used equally in a species [2], [3] and the most frequently used synonymous codons correspond to the most abundant tRNAs [4], [5] in a species. The fraction of optimal codons in highly expressed genes becomes much higher than in the usual genes and hence the codon usage bias was thought to regulate translation efficiency [6], [7]. Sharp and Li formulated the codon bias as RSCU (Relative Synonymous Codon Usage) and proposed an index (CAI, Codon Adaption Index) to reflect the strength of the bias in specific genes of a genome [8]. CAI is a proxy of expression level of genes and it could be raised through optimizing codon usage [9]. Such a method has been well accepted as the basic biotechnical approach of enhancing expression level of exogenous genes [10], [11], [12]. Aided with the high-output technology, recent genome-scale investigations re-exhibited the association of codon bias and expression level and hence confirmed the reasoning of translation efficiency selection [13], [14], [15], [16], [17]. These latest larger-scale researches involved expression level data including microarray, RNA-seq and mass spectrum proteome, which in fact reflected the translation efficiency from mRNA to proteins [18], [19].

On the other hand, a few pioneer researchers paid attention to the association between codon identity and amino acid property. Crick [20] was the first to observe that all codons with uracil (U) in the second place coded for hydrophobic amino acids. Later Taylor and Coates re-noted [21] the link between the middle codon position and the hydrophobicity- hydrophilicity spectrum. With the reliable measure of hydropathy, they observed that five of the six extremely hydrophobic amino acids have U as the middle base. Nearly all of the extremely hydrophilic amino acids have adenine (A) in this site, whereas the neutral amino acids have guanine (G) or cytosine (C). Crick [20] explained that this link is caused by the base-amino acids affinity (or stereo-chemical fit) and Yarus *et al.* [22] explained it as the result of evolutionary adaptation. It was thought that the two alternative explanations could be compatible [22]. Pieces of evidence have been proposed to support these mechanistic explanations (stereo-chemical fit) [23], [24], [25] or theoretical speculations (selection constraints) [26], [27].

However, there is a shortage of systematic investigation between codon position and amino acid physicochemical properties. One of the open questions is that whether there are links between the other physicochemical properties and specific codon positions. Therefore, here we proposed a quantitative measurement of codon-amino acid association and used it to explore 13 most frequently mentioned properties of amino acids.

## 2 Material and methods

### 2.1 Genomic data download

We downloaded genomic data of prokaryotes from NCBI on March 26th 2017, with a total of 2774 genomes (ftp://ftp.ncbi.nlm.nih.gov/genomes/archive/old_refseq/Bacteria/). Then we removed the plasmids, fragments within some genomes and retained the coding genes sharing in the three types of files. After the unqualified data was removed, we used the remaining 2764 prokaryotic species for further study. (.ffn is FASTA nucleotide coding regions file, .faa is FASTA amino acid file, .ptt is protein table file).

We downloaded genomic data of eukaryotes, including 68 metazoa, 186 protists, 735 fungi and 44 plants from https://asia.ensembl.org/downloads.html. Furthermore, we downloaded *Homo sapiens* genome from http://ftp.ensemblorg.ebi.ac.uk/pub/release-97/fasta/homo_sapiens/ and *Mus musculus* genome from http://ftp.ensemblorg.ebi.ac.uk/pub/release-97/fasta/mus_musculus/.

In total, we have 2764 + 68 + 186 + 735 + 44 + 2 = 3799 genomes to investigate.

### 2.2 Single nucleotide combinations and assigning their values

Our proposed quantitative measure begins with the two major steps of producing nucleotide combinations and then assigning specific values to them. Codons are non-overlapping triplets and each of the three positions has four nucleotide candidates. When we study the association of codon with amino acids’ properties, at first, we combine four nucleotides at each position. There are three general combination types, i.e., four nucleotides into four groups, three groups, and two groups separately. When all four nucleotides are taken as one group, we could not find any meaningful link between them and amino acids.

In detail, there is only one four-group combination ($C_{4}^{4}$) with each of the four nucleotides in one group. For the three groups, base A may combine with G, or C, or T, alternatively, G with C or T, and the last combination is C with T. Therefore, there are six $\left(C_{4}^{2}\right)$nucleotide combinations for the form of three-group. For two-group combination, we can set only one nucleotide in one group and the other three nucleotides into the second group, the number of combinations in this case is $\left(C_{4}^{1}\right)$. Alternatively, two nucleotides in one group and the other two nucleotides in the second group. In this case, the combination number is ($C_{4}^{2}$).

To quantitatively measure the association between nucleotide combinations and property, we need to assign values to each specific group in one specific combination. Taking the three-group AG-C-T as an example, we could assign the values as (AG: −1) – (C: 0) – (T:1), which means if one amino acid has A or G at this position, then the nucleotide value is −1. As for the only one four-group combination, we have$P_{4}^{4}$ ways of assigning values. For each of the six three-group combination, it has $P_{3}^{3}$ways of assigning values. In the former case of two-group (four combinations), the number of ways to assign values for each combination is $P_{2}^{2}$, and in the latter case (six combinations), the number of ways to assign values would be $P_{2}^{2}Ã\cdot 2$or $C_{2}^{2}$.

The above two-step procedure is applicable to each codon position. Note that there are three codon positions. Hence, as an example, the number of four-group nucleotide permutation after assigning group values should be $C_{4}^{4}{\times P}_{4}^{4}\times 3=72.$Similar calculation would be obtained for three and two-group permutations.

### 2.3 Values of physicochemical properties of amino acids

Amino acids are needed to assign values, but it is more convenient than nucleotides because all physicochemical properties have their numerical order for 20 amino acids. Here we extracted a total of 13 physicochemical properties Table 1, which are all well studied.

**Table 1:** Property list and associated references.

Property number	Property names	Reference
3	molecular weight, melting point, isoelectric point	[28]
1	hydropathy index	[29]
3	chemical composition of the side chain, molecular volume, polarity	[30]
1	refractivity	[31]
4	aromaticity, aliphaticity, hydrogenation, hydroxythiolation	[32]
1	polar requirement	[33]

We would like to note that if a researcher is interested in any other properties not included here, he or she could make a similar correlation analysis for his or her interested properties.

With the two steps of combining nucleotides and assigning values, for each codon position and each property, there will be $C_{4}^{4}{{\times P}_{4}^{4}+C_{4}^{2}\times P_{3}^{3}+{(C}_{4}^{1}{\times P}_{2}^{2}+C}_{4}^{2}{\times C}_{2}^{2})=24+36+\left(8+6\right)=74$ associations. The total number of involved associations will be 74×3×13=2886.Note that these are the candidate associations and next we will use quantitative correlation analysis to choose out the highest association for each codon position and each property.

### 2.4 Quantitative association between nucleotide combination and physicochemical property

Here, we used the Pearson correlation coefficient (PCC) to measure the degree of linear correlation between the two variables of nucleotide combination and amino acid property [34]. The correlation coefficient R ranges from −1 to 1. The positive or negative values discriminate the direction of correlation and the higher absolute value means the higher extent of correlation.

In this study, we used the PCC in two places: one is to measure the association between single nucleotide combination and 13 physicochemical properties of amino acids and the other is to analyze the relationship between the relative frequencies of base T and A at the second codon position and the GRAVY score of proteins.

GRAVY score is produced by the CodonW software and we download it from http://codonw.sourceforge.net/#Downloading%20and%20Installation (Last updated 7/May/2005 by John Peden). There are instructions for users to calculate the GRAVY score of proteins, which is representative hydrophobicity of protein. It is calculated as the arithmetic mean of the hydropathy index of all amino acids in a specific protein.

### 2.5 Informational genes versus operational genes

We used COG [35] and KOG (eukaryotic orthologous groups) [36] framework to reflect functional category distinction. Here, we obtained COG and KOG dataset from https://www.ncbi.nlm.nih.gov/research/cog-project/ to annotate gene function of each genome (six representative species of three domains: *Escherichia coli* and *Bacillus subtilis* for bacteria, *Methanococcus jannaschii* and *Halobacterium* NRC 1 for archaea, *Saccharomyces cerevisiae* and *Homo sapiens* for eukaryotes). Each data set contains information on each orthologous group, including the group name, functional category letter, function description, and the list of proteins. There are 26 letters to represent the 26 function categories for both COG and KOG. COGs with the codes (A, B, J, K, L) belong to the information super-class and the codes (L, D, M, N, O, T, U, V, W, X, Y, Z, C, E, F, G, H, P, Q, R, S) fall to the operation super-class.

### 2.6 Subcellular location of informational genes versus operational genes

To reveal the effect relevant with difference of hydrophobicity between informational proteins and operational proteins, we checked the subcellular location of two groups of proteins by the PSORTdb4.0 (https://db.psort.org/) [37], which is a database of protein subcellular localizations for bacteria and archaea and contains both information determined through laboratory experimentation (ePSORTdb dataset) and computational predictions (cPSORTdb dataset). We adopted the *E. coli* str. K-12 substr MG1655 data verified by laboratory experimentation.

### 2.7 Statistical analysis of GRAVY score and T_2_-A_2_ difference between two groups of proteins (genes)

We used the student *t*-test [34] to check the significance of the difference between two groups of proteins (genes).

The independent (unpaired) samples *t*-testis is used to analyze GRAVY and T_2_-A_2_ difference in six model species.

### 2.8 Statistical analysis of subcellular location difference between informational proteins and operational proteins

A chi-squared test, also written as x^2^ test, is used to determine whether there is a significant difference between the expected frequencies and the observed frequencies of subcellular location types in two groups of proteins [34]. Hence, a 2 × 2 chi-square table is constructed.

## 3 Results

### 3.1 Quantitative association between nucleotide combination and 13 physicochemical properties

We try to quantitatively elucidate the association between codon position and amino acids. Using the two-step method, we checked each combination of single nucleotides at all three codon positions with each of the 13 physicochemical properties. The complete catalogue is listed in Table S1. Four nucleotides could be divided into four-group, three-group and two-group combinations.

To help grasp the schematic information from the complete 2886 candidate associations, we only chose the highest association for each codon position and each property. For the 39 strongest associations, we compiled the Table 2. As can be seen from it, seven (polar requirement, aliphaticity, hydrogenation, chemical composition of the side chain, molecular volume, polarity, hydropathy index) out of 13 properties have highest associations with the second codon position and the hydropathy index is the most notable one. Furthermore, the average correlation coefficient of the second codon position is higher than that of the other two sites. Crick and followers have observed a quantitative relation between the second codon position and the hydrophobicity [20], [25]. Here we illustrated that there are six other properties associated with the second codon position more than with the other two codon positions based on the quantitative measurement. This result indicates that the proteins’ structure and function would be determined by the middle codon position more than the other two sites, especially the general function of proteins. In other words, the second codon position would contain more information of protein function than the other two positions.

**Table 2:** Pivotal information from the catalogue of the associations between single nucleotide combination and 13 properties.^a^

Property	First position			Second position			Third position			General^b^		
	Combination	R1	P1	Combination	R2	P2	Combination	R3	P3	R	R^2^	P
hydropathy index	T:1, C:-1, AG:0	0.352	1.30E-01	A:-1, T:1, GC:0	**0.864**	9.40E-07	A:-1, T:1, GC:0	0.153	5.20E-01	0.891	0.793	1.00E-05
polarity	T:-1, CAG:1	0.542	1.40E-02	A:1, T:-1, GC:0	**0.788**	3.70E-05	A:1, T:0, GC:-1	0.338	1.40E-01	0.904	0.817	3.90E-06
hydrogenation	T:0, G:1, AC:-1	0.175	4.60E-01	A:-1, C:1, TG:0	**0.767**	8.00E-05	A:1, G:-1, TC:0	0.094	6.90E-01	0.799	0.638	8.00E-04
polar requirement	T:-1, G:1, AC:0	0.56	1.00E-02	A:1, T:-1, GC:0	**0.73**	2.60E-04	A:2, T:-2, G:-1, C:1	0.31	1.80E-01	0.87	0.757	3.60E-05
refractivity	T:1, G:-1, AC:0	**0.712**	4.30E-04	G:1, C:-1, AT:0	0.518	1.90E-02	A:-1, T:0, GC:1	0.446	4.90E-02	0.812	0.66	5.00E-04
aliphaticity	A:1, G:-1, TC:0	0.41	7.30E-02	T:1, CAG:-1	**0.631**	2.90E-03	T:-1, G:0, AC:1	0.197	4.10E-01	0.749	0.561	3.60E-03
aromaticity	T:1, C:0, AG:-1	**0.587**	6.50E-03	A:0, C:-1, TG:1	0.282	2.30E-01	A:-1, T:0, GC:1	0.407	7.50E-02	0.637	0.406	3.60E-02
cc of the side chain c	T:1, G:0, AC:-1	0.168	4.80E-01	A:1, T:-2, G:2, C:-1	**0.573**	8.30E-03	A:-1, T:1, G:-2, C:2	0.354	1.30E-01	0.673	0.453	1.90E-02
hydroxythiolation	T:1, CAG:-1	0.451	4.60E-02	G:0, C:1, AT:-1	0.353	1.30E-01	T:1, G:0, AC:-1	**0.562**	9.90E-03	0.694	0.481	1.30E-02
molecular weight	T:1, G:-1, AC:0	**0.539**	1.40E-02	C:-1, GAT:1	0.485	3.00E-02	A:-2, T:1, G:2, C:-1	0.432	5.70E-02	0.74	0.548	4.50E-03
isoelectric point	A:1, T:-1, G:-2, C:2	**0.536**	1.50E-02	G:1, CAT:-1	0.253	2.80E-01	A:1, T:-1, GC:0	0.276	2.40E-01	0.671	0.45	2.00E-02
molecular volume	T:1, G:-1, AC:0	0.512	2.10E-02	T:1, C:-1, AG:0	**0.527**	1.70E-02	A:-2, T:1, G:2, C:-1	0.373	1.10E-01	0.731	0.534	5.70E-03
melting point	A:-1, T:2, G:1, C:-2	0.341	1.40E-01	T:1, G:0, AC:-1	0.427	6.00E-02	A:-1, TGC:1	**0.527**	1.70E-02	0.652	0.425	2.80E-02

a These properties are listed in the descending order of the highest R value among three codon positions. For each of the 13 properties the codon position with the highest correlation is marked as bold fonts. As can be seen, the second codon position has seven top values, whereas the first and the third codon position only have four top and two top correlations respectively. Among the 39 optimal combinations, you can see four nucleotides could be classified into two, three or four groups.

b This column calculated the combined association between all three codon positions with each property.

c Chemical composition of the side chain, i.e., the atomic weight ratio of hetero or noncarbon elements in end groups or rings to carbons in the side chain.

If we aggregate the three codon positions together, then eight properties (polar requirement, aliphaticity, hydrogenation, molecular volume, polarity, refractivity, hydropathy index, molecular weight) have square R higher than 0.5 (Table 2), indicating single nucleotides could generally determine their values. For the other five properties, their values would be majorly determined in the form of adjacent di-nucleotides.

We want to explain the regularities revealed here and take the second highest association as an example. It appears between the second codon position and polarity. As you can see from Table 2, the nucleotide combination is (A:1, T:-1, GC:0), which means that amino acids with nucleotide A at the second codon position has the highest polarity value, T with the slowest polarity value at this site, and G or C with the neutral value. Similarly, the highest association for the first codon position exists with refractivity, which means amino acids with T at this position has the highest refractivity, amino acids with G at this site has the lowest refractivity and A or C has medium refractivity.

### 3.2 Strong association between the hydrophobicity of proteins and the relative frequencies of base T and A at the second codon position

The single nucleotide combination (A_2_: −1, T_2_: 1, G_2_C_2_: 0) and hydropathy index exhibit the highest association among all pairs of variables. This means if we assign the value of 1 to nucleotide T and the value of −1 to nucleotide A and 0 to both G and C at the second codon position (three-group combination), then it could get the association coefficient R of 0.864. Note that we assign different values to four nucleotides just to maximize the correlation obtained. If we assign all of them with the same value, we could not detect any association signal. Such assigning values of A_2_: −1, T_2_: 1, G_2_C_2_: 0, means amino acids with A at the second codon position has the lowest hydropathy index, T has the highest index, whereas G or C with the medium hydropathy.

**Fig. 1:**
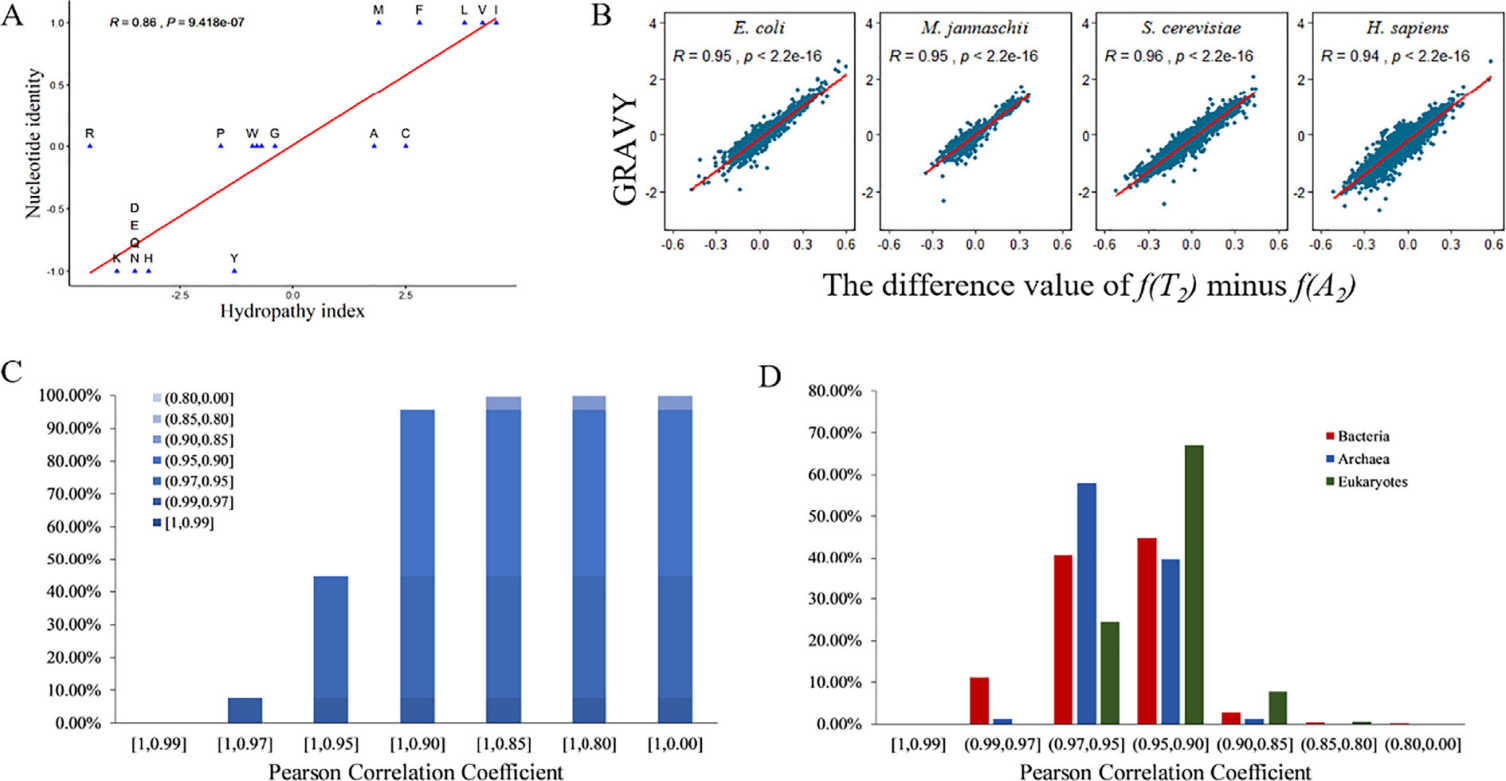
Amino acid level correlation amplifies at protein level between nucleotide identity and hydropathy index. (A) The scatter plot of hydropathy index of 20 single amino acids and nucleotide identity at the second codon position. (B) Four representative examples illustrate the strong association between T_2_-A_2_ frequencies and GRAVY values of all genes (proteins). (C) Cumulative proportion plot of linear correlation coefficient R. As you can see, 95.5% species have R value higher than 0.9 (p < 1e-60) and 44.8% species higher than 0.95 (p < 4e-75, Table S2). (D) Distribution of correlation coefficient (statistical histogram) R in each of the three domains. Note that all the peaks appear in the range of [0.9, 0.95].

This above association is measured at the level of codon and could be visually shown in Fig. 1A. Then we aggregate the effect of all codons in each gene, that is to say, we could give all T nucleotides at the second codon position (T_2_) a value of 1, A of −1, and G_2_ or C_2_ of 0. When aggregating all nucleotides at this codon position, the contribution of G_2_ and C_2_ to the gene will be neglected because of their zero values. Therefore, at the scale of gene, the optimal single nucleotide effect would be simplified as T_2_-A_2_, i.e., the relative frequencies of nucleobase T and A at the second codon position.

On the other hand, we obtained the GRAVY value (the global hydrophobicity value of proteins) using the codon tool of all proteins in 3799 genomes, including 2600 bacteria, 164 archaea, and 1035 eukaryotes. Next, we calculated the association coefficient of T_2_-A_2_ frequencies and GRAVY scores for each genome. All four representative genomes from bacteria (*E. coli*), archaea (*M. jannaschii*), single cell eukaryotes (*S. cerevisiae*) and higher eukaryotes (*H. sapiens*) (Fig. 1B), show very strong associations (R≈0.95), which are much higher than that in Fig. 1A, demonstrating an amplifying effect at the protein level compared to single amino acid level. Taking protein as the studying unit will significantly enlarge the size of samples than single amino acids and in the latter case there are only 20 elements (samples). Hence the correlation coefficient could be amplified and this may be regarded as a population effect.

Taking all 3799 genomes as a collective (Table S2), the cumulative histogram shows that 95.5% (3629) genomes have R > 0.9 and simultaneously their p-value < 1e-60. Furthermore, near perfect association exists in 44.8% (1702) genomes (R > 0.95 and p < 1e-74). And only two (2/3799≈0.053%)genomes have R < 0.80 (R = 0.73 and 0.76, respectively). Both the two exceptions belong to the same species *Candidatus tremblaya*, *C. tremblaya* is endosymbiont of eukaryotic cells and has the smallest gene numbers (121 and 116 genes, respectively) in all the investigated genomes. We think such low gene number reduces the value of association coefficient. Taken together, we could definitely say R for all genomes is larger than 0.73 and square R is larger than 0.53. In fact, if we filter the 43 genomes with gene number less than 500, then the minimum R will be 0.81 and the average R will be 0.94.

If we classify 3799 genomes into three domains (bacteria, archaea and eukaryotes), it can be found that the peak of R value in the histogram all appear in the interval of 0.9–0.95 (Fig. 1D), indicating a generally strong association for each of the life domains. Hence, proteins’ hydrophobicity is almost completely determined by the frequency of T_2_-A_2_. This has built up a connection between codon and protein’s property and this link is universal and independent of species and life domains.

### 3.3 Informational function holding proteins have lower GRAVY scores (higher hydrophilicity)

It is natural to ask whether the association pattern of T_2_-A_2_ and hydrophobicity influences proteins’ functions. Informational and operational function holding proteins (genes) are the two basic functional super-categories in three life domains [38]. Here we extracted such classifying information for six representative genomes and compared their hydrophobicity by the proxy of GRAVY within two groups of proteins. The former codes for functions of genetic information producing and transferring. The latter encodes the rest of the functions, such as metabolism, transduction and regulation.

As can be seen from Table 3, there are two widely-studied species for each domain. Without exception, the informational function holding proteins in all six species have significantly lower GRAVY score than the operational function holding proteins (all p < 3e-37). For example, in *E. coli***,** the mean GRAVY score is −0.291 for the 693 informational function holding proteins, whereas the 3236 operational function holding proteins have the higher mean GRAVY score of −0.006 and the p-value of *t*-test is 1.084e-62. In *S. cerevisiae*, the 1061 informational function holding proteins have the mean GRAVY score of −0.551, whereas the 3298 operational function owing proteins have the mean GRAVY score of −0.336 (Table S3). Obviously, informational function holding proteins are more hydrophilic. Similar patterns are observed in the other four species. Hence, the link between proteins’ GRAVY score and their general functions is universal and is independent of life domains.

**Table 3:** GRAVY and T_2_-A_2_ test between two groups of proteins (genes) in six model species.

Domains	Species	GRAVY test^a^			T_2_-A_2_ test^b^		
		informational	operational	P-value	informational	operational	P-value
bacteria	*E. coli*	−0.291	−0.006	1.084E-62	−0.025	0.025	1.476E-31
	*B. subtilis*	−0.392	−0.073	2.216E-56	−0.073	−0.006	4.637E-38
archaea	*M. jannaschii*	−0.374	−0.061	3.084E-37	−0.076	−0.022	3.875E-18
	*Halobacterium* NRC 1	−0.440	−0.065	2.166E-53	−0.074	0.003	2.612E-38
eukaryotes	*S. cerevisiae*	−0.551	−0.336	1.406E-65	−0.101	−0.059	5.819E-43
	*H*. *sapiens*	−0.537	−0.324	1.880E-59	−0.082	−0.041	1.155E-37

a We calculated the average GRAVY score of all informational function holding proteins and that of all operational function holding proteins, then used the student *t*-test to check the significance.

b We calculated the average T_2_-A_2_ frequency of all informational function encoding genes and that of operational function encoding genes, then a student *t*-test was carried out.

We also found that T_2_-A_2_ values have the consistent difference between the two groups of genes in all six species, with p-values < 3e-18 (Table 3). Hence, the strong association between GRAVY score and T_2_-A_2_ is further validated at the genomic level (Protein group with lower GRAVY score will have lower T_2_-A_2_ frequency). This result just illustrates our above proposal: the second codon position contains more functional information.

3.4. Potential explanations for the difference of hydrophobicity between informational and operational function holding proteins

Then we try to reveal potential reasons or relevant factors for the observation that informational function holding proteins have higher hydrophilicity than operational function owning proteins. We checked the subcellular location of two protein groups (informational and operational) in *E. coli*. According to the subcellular location database of prokaryotes PSORTdb4.0 (https://db.psort.org/) [37], a total of 745 proteins have validated location information, among which 681 have operational functions and the rest 64 perform informational functions. These proteins are assigned to one of two subcellular locations, i.e., cytoplasmic membrane and cytoplasm. For the informational function holding proteins, 98.4% are located at the cytoplasmic environment, whereas 75.5% operational function holding proteins lie on the cytoplasmic (Table S4). A Chi-square test indicates the significant difference in location type between the two groups of proteins (p < 0.0001), which means the informational and operational functions holding proteins are distinct in subcellular locations. Cytoplasmic environment contains more water than cytoplasmic membrane [39], [40] and this may constitute the underlying reason why informational function holding proteins have lower GRAVY score (higher hydrophilicity).

On the other hand, Table 3 illustrates that T_2_-A_2_ frequencies have a consistent difference between informational function encoding genes and operational function encoding genes with GRAVY score in all six species. Such consistency could be considered as the factor of mutational mechanism or underlying reason. Note that there should exist some other explanations of the GRAVY score difference between two groups of genes. However, we give plausible ones from both adaption selection and neutralist viewpoints. We think it may be possible to interpret most evolutionary events from both internal and external causes and often they are not contradictory.

## 4 Discussion

Pioneer biologists have visually observed scattered associations between single nucleotide identity and a few properties of amino acids from the codon table [20]. Here, we first compiled a catalogue of all single nucleotide combinations and a total of 13 physicochemical properties based on quantitative measurements. From the extracted Table 2, we found that seven among the 13 properties have the highest associations with the second among three codon positions. Previously, it was found that the first base of codons strongly associates with the precursor from which the encoded amino acid is synthesized [41], and the third codon position denotes the degenerate site and strongly influences genes’ expression level [7], [8]. Here, we systematically revealed the second codon position determines most physicochemical properties of amino acids and hence would majorly determine the protein’ structure or function, especially the general function. Consistently, evolutionary researchers have proposed that because the second codon position most determined the identity of amino acids, this site should be more conserved than the other two sites [42].

When pioneer scientists observed the association between the second codon position and the hydropathy, they only got a qualitative association and did not measure its strength [20], [21]. Here, by combining our proposed method of value assignment to nucleotide combination and the often-used Pearson correlation analysis, we obtained quantitative strength of this association. Furthermore, we checked the relationship between each codon position and each of the 13 properties and chose those most significant associations. Although our newly revealed associations are not as strong as the hydropathy-middle position association, they indeed are much statistically significant. Only with them, could the association information between specific codon position and amino acid property be regarded as complete.

Protein function would depend on DNA sequence [43]. However, proofs are needed to provide support for this judgement. Experimentally, if we mutate the vital sites of DNA sequence then its coding protein would lose the natural functions [44]. As a complement, here we computationally described an example illustrating the complete connection from codon to amino acid and then to proteins’ general function. The link between genes’ T_2_-A_2_ frequencies and proteins’ GRAVY score (hydrophobicity) is generally strong and universally appears in all the 3799 genomes investigated. The GRAVY score difference is compared for six most well-studied genomes of three domains, and informational function holding proteins are found to have higher hydrophilicity than operational proteins. This result indicates a link between amino acids’ property and proteins’ general functions. The two connected results may help us understand how DNA sequence determines protein functions.

## 5 Conclusions

In this study, we proposed a quantitative measurement of codon-amino acid association and used it to explore 13 physicochemical properties of amino acids. Consequently, seven properties have a higher correlation with the middle position than the other two sites, indicating its major role in determining proteins’ functions. At the protein level, the correlation between the frequency of A_2_ relative to T_2_ and the hydrophobicity score becomes stronger than the single amino acid level. All 3799 involved genomes of three domains have regression coefficient *R* higher than 0.73, indicating the universal appearance of such association. Finally, it was observed that informational function holding proteins have lower GRAVY values than operational proteins and this difference may be relevant to the subcellular location. Altogether, a complete link from codon identity to amino acid property and then to protein functional categories is revealed.

## Author contributions

F-BG designed and coordinated this project. Y-TJ did the computation work. T-YJ, Z-LZ and Y-NY double checked the results. F-BG and Y-TJ analyzed the results and drafted the manuscript. JW and F-B Guo revised the manuscript with comments from other authors.

## Declaration of Competing Interest

The authors declare that they have no known competing financial interests or personal relationships that could have appeared to influence the work reported in this paper.

## Appendix A Supplementary data

Supplementary Table 1 Pearson correlation coefficient between nucleotide combination and physicochemical property.

Supplementary Table 2 Pearson correlation coefficient between the Hydrophobicity of Proteins and the Relative Frequencies of Base T and A at the Second Codon Position (1).

Supplementary Table 3 Informational proteins and operational proteins in six model species.

Supplementary Table 4 Subcellular location of informational proteins and operational proteins.
